# Antigen Format Determines Immunogenicity of AAV-Based SARS-CoV-2 Vaccines: Full-Length Spike Versus Truncated Subunits

**DOI:** 10.3390/vaccines13121187

**Published:** 2025-11-24

**Authors:** Anna V. Vakhrusheva, Maria E. Frolova, Arthur A. Isaev

**Affiliations:** 1 Betuvax JSC, Moscow 119571, Russia; 2 Swiftgen LLC, Moscow 119571, Russia; frolova@nextgene.ru; 3 Artgen Biotech PJSC, Moscow 119571, Russia; art.isaev@hsci.ru

**Keywords:** AAV-based vaccines, adeno-associated virus (AAV9), SARS-CoV-2, COVID-19, spike protein, S1 subunit, receptor-binding domain (RBD), vaccine design

## Abstract

Background: Antigen format strongly influences the immunogenicity of gene-based vaccines. Full-length Spike is widely used in licensed COVID-19 vaccines, while truncated subunits such as S1 or the receptor-binding domain (RBD) may simplify vector design but risk reduced potency. We aimed to compare these antigen formats in an AAV9 delivery platform. Methods: BALB/c mice were immunized intramuscularly with recombinant AAV9 encoding full-length Spike, S1, or RBD at doses of 1 × 10^10^ or 1 × 10^11^ viral genomes. Immune responses were assessed by serology, virus neutralization, T-cell profiling, and histopathology. Results: All constructs expressed antigen in vitro and in vivo. Only full-length Spike elicited robust neutralizing antibodies at both doses, with titers rising significantly by week 12. High-dose RBD induced neutralization in a minority of animals, whereas S1 failed to do so. Antigen-specific IgG responses scaled with insert length (Spike > S1 > RBD). Cellular immunity was dominated by CD8^+^ effector memory T cells, strongest in the Spike group, which also induced measurable CD4^+^ responses. Local transient myositis was observed at the injection site but resolved by week 24, with no systemic pathology. Conclusions: Full-length Spike outperforms truncated subunits in the AAV context, highlighting antigen structure as a critical factor for next-generation coronavirus vaccine design.

## 1. Introduction

SARS-CoV-2, a member of the Coronaviridae family, is the causative agent of COVID-19. The 2020–2023 pandemic has become one of the deadliest in recent history, with over 7 million reported deaths and ~775–780 million confirmed cases globally to date [1]. Although the World Health Organization (WHO) ended the Public Health Emergency of International Concern (PHEIC) on 5 May 2023 [2], COVID-19 remains an ongoing global health issue. Current WHO surveillance indicates continued SARS-CoV-2 circulation worldwide; for example, in the week 25–31 August 2025, 2375 of 44,580 tested samples (5.3%) were positive across 70 reporting countries [1]. These data represent global population-level statistics reported by the WHO COVID-19 Dashboard and are not limited to hospitalized patients.

From the outset of the pandemic, the urgent need for safe, effective, and scalable vaccines was clear. Various platforms were employed in vaccine development, including inactivated and live-attenuated viruses, recombinant proteins, and nucleic acid-based approaches such as mRNA and DNA vaccines. The development of COVID-19 vaccines has involved strategic selection of different SARS-CoV-2 antigens across various vaccine platforms, with each approach utilizing distinct portions and configurations of the Spike protein to elicit protective immune responses.

By 2024, more than 64 vaccines against COVID-19 had been approved globally [3], with the majority targeting the full-length Spike (S) protein of SARS-CoV-2—a trimeric surface glycoprotein essential for viral entry via binding to the ACE2 receptor [4,5]. Most widely used vaccines, including Pfizer/BioNTech’s BNT162b2, Moderna’s mRNA-1273 [6,7], Oxford–AstraZeneca’s ChAdOx1 nCoV-19, and the Russian Sputnik V and single-dose version Sputnik Light [8,9,10,11], encode the full-length prefusion-stabilized Spike protein. In contrast, some recombinant protein and subunit vaccines, such as Betuvax-CoV-2, use truncated forms of Spike [12]. A particularly important region within the S1 subunit is the receptor-binding domain (RBD), which mediates attachment to host cells and serves as a major target for neutralizing antibodies [13]. Notably, subunit vaccines encoding only the RBD or the entire S1 domain have demonstrated robust immunogenicity and protective efficacy in both preclinical and clinical studies [12, 14,15,16,17]. These examples illustrate that the choice of antigen format could vary by platform and directly affects immunogenicity and production feasibility.

A major challenge for all vaccine platforms is the ongoing antigenic evolution of SARS-CoV-2. Variants such as Delta and Omicron have accumulated mutations in key regions of the Spike protein, leading to reduced vaccine efficacy and partial immune escape [18,19,20,21,22,23]. In response, updated formulations of existing vaccines have been introduced, including reformulated mRNA boosters [24] and updated adenovirus-based vaccines such as the reformulated single-dose Sputnik Light.

These developments highlight the importance of adaptable and durable vaccine platforms. In contrast to these adenoviral platforms, adeno-associated virus (AAV) vectors offer several unique advantages for vaccine development: they enable long-term antigen expression from non-replicating episomal DNA, have better safety profile established in gene-therapy applications, and typically induce only mild local reactogenicity. Moreover, AAV particles are highly stable and can be stored for extended periods without loss of activity [25]. Although pre-existing and induced anti-capsid antibodies can limit redosing, alternate or engineered serotypes may, under certain conditions, facilitate re-administration. These features make AAV an attractive vector for next-generation, durable gene-based vaccines [26].

The immunogenicity of gene-based vaccines is determined by multiple structural and expression-related parameters of the encoded antigen, including its length, folding stability, membrane anchoring, glycosylation pattern, and epitope accessibility. Larger or membrane-anchored antigens typically provide a broader epitope repertoire and more efficient presentation to B and T cells, whereas small soluble domains may exhibit reduced persistence and faster clearance. These structural factors can profoundly influence both the magnitude and quality of the immune response. Therefore, it remains unclear which antigen format—full-length Spike, truncated S1 subunit, or minimal RBD—offers the optimal balance between immunogenicity, durability, and breadth of protection when delivered via AAV. To address this, we designed and evaluated three AAV-based vaccine constructs encoding (i) the full-length Spike protein carrying mutations representative of the Beta variant, (ii) the S1 subunit, and (iii) the RBD alone. The full-length Spike was selected to preserve the native trimeric conformation and membrane anchoring, the S1 subunit to represent the major receptor-binding region while omitting the transmembrane and S2 domains, and the RBD to serve as a minimal antigenic unit containing key neutralizing epitopes. Using a mouse model, we systematically compared the humoral and cellular immune responses elicited by each construct following intramuscular administration.

## 2. Methods and Materials

### 2.1. Reagents and Materials

Plasmids pAAV-CMV, pHelper, and pAAV-RC2/9 were from the in-house collection of Marlin Biotech, Moscow, Russia.

#### 2.1.1. Constructs and Plasmids

SARS-CoV-2 gene fragments (full-length S, S1, and RBD), and primers were synthesized by Evrogen, Moscow, Russia (Table S1). Plasmid maps are available upon request. Full coverage of the expression cassette was achieved for each plasmid. No significant mutations were identified.

#### 2.1.2. Bacterial Strains and Cloning Reagents

*E. coli Stbl3* cells were purchased from Thermo Fisher Scientific, Waltham, MA, USA (Cat. No. C737303). LB broth (Cat. No. L3022), agar (Cat. No. A7002), and kanamycin monosulfate (Cat. No. K1377) were obtained from Sigma-Aldrich, Saint Louis, MO, USA. Linear polyethylenimine (PEI, MW 25,000) was purchased from Polysciences, Inc., Warrington, PA, USA (Cat. No. 23966).

#### 2.1.3. Cell Culture

HEK293FT cells were obtained from Thermo Fisher Scientific, Waltham, MA, USA (Cat. No. R70007) and cultured in DMEM/F12 (1:1) medium supplemented with 10% fetal bovine serum (FBS), 1 mM sodium pyruvate, 1% GlutaMAX, and 1% antibiotic–antimycotic solution (all from Gibco, Waltham, MA, USA). Vero cells were obtained from ATCC, Manassas, VA, USA (Cat. No. CCL-81) and maintained under standard conditions.

#### 2.1.4. Viral Particles

Inactivated SARS-CoV-2 Beta variant virus (strain hCoV-19/Russia/SPE-RII-27029V3/2021, GISAID ID: EPI_ISL_8112507) and SARS-CoV-2 Delta variant virus (strain hCoV-19/Russia/SPE-RII-32759V3/2021, GISAID ID: EPI_ISL_8112510) were provided by Vector-Best, Novosibirsk, Russia and used in experiments conducted in biosafety level II containment facilities.

#### 2.1.5. Western Blot Reagents and Antibodies

Primary antibodies XR-06 and XR-25 (anti-RBD) were purchased from Xema Medica, Moscow, Russia (catalog numbers unavailable). Goat Anti-Mouse IgG H&L (HRP-conjugated) secondary antibody was purchased from Abcam, Cambridge, UK (Cat. No. ab97040). Clarity™ Western ECL substrate was obtained from Bio-Rad, Hercules, CA, USA (Cat. No. 1705060).

#### 2.1.6. Reagents for T-Cell Stimulation Assays

PepTivator^®^ SARS-CoV-2 Prot_S peptide pool was obtained from Miltenyi Biotec, Bergisch Gladbach, Germany (Cat. No. 130-126-701). Anti-mouse CD28 costimulatory antibody was purchased from BioLegend, San Diego, CA, USA (Cat. No. 102105). Phorbol 12-myristate 13-acetate (PMA) was purchased from Sigma-Aldrich, Saint Louis, MO, USA (Cat. No. 50-165-6915). Ionomycin was purchased from Sigma-Aldrich, Saint Louis, MO, USA (Cat. No. I0634).

### 2.2. Animal Experiments

For in vivo experiments, female BALB/c mice (6–8 weeks old) were used. The animals were obtained from the Stolbovaya branch of the Scientific Center of Biomedical Technologies, Federal Medical and Biological Agency of the Russian Federation, Moscow, Russia.

Mice were housed in sterile polycarbonate cages with wood pellet bedding, under specific pathogen-free (SPF) conditions. Animals had free access to standard chow and autoclaved drinking water (ad libitum). Environmental parameters were maintained as follows: temperature 18–24 °C, relative humidity 50–80%, and a 12 h light/dark cycle.

All animal procedures were conducted in accordance with national and institutional guidelines and were approved by the Ethics Committee of the Smorodintsev Research Institute of Influenza, Saint Petersburg, Russia (protocol No. 02, dated 24 January 2023).

### 2.3. Design, Construction, and Preparation of AAV9-Based Vaccines

The sequences of the plasmids pAAV-SARS1-5, encoding various SARS-CoV-2 antigens, were cloned into the pAAV-CMV expression vector (Marlin Biotech, Moscow, Russia) using ligation-independent cloning, according to a standard three-plasmid AAV production system protocol. Chemically competent *E. coli Stbl3* cells (Thermo Fisher Scientific, Waltham, MA, USA) were transformed with the recombinant plasmids and cultured on LB agar plates containing kanamycin (50 µg/mL). Plasmid DNA was isolated from positive colonies using a standard alkaline lysis miniprep protocol.

Insert identity and orientation were verified by restriction enzyme digestion and Sanger sequencing, primers are presented in Table S2. Specific enzyme combinations were used for each construct:

• pAAV-SARS1 (Spike Wuhan): digested with NheI and BbsI, yielding fragments of 1774 bp, 2569 bp, and 4046 bp.
• pAAV-SARS2 (Spike Beta) and pAAV-SARS3 (S1 Beta): digested with MluI (single site), yielding linear fragments of 5059 bp and 5379 bp, respectively.
• pAAV-SARS4 (RBD Beta): digested with BstBI and MluI, yielding fragments of 1289 bp and 3725 bp.
• pAAV-SARS5 (Spike Delta+): digested with EcoRV, yielding three fragments of 617 bp, 2510 bp, and 4169 bp.

The restriction profiles matched theoretical predictions for each plasmid (Figure S1), confirming successful cloning of the SARS-CoV-2 antigen sequences.

### 2.4. Production and Purification of Recombinant AAV9

For large-scale production of AAV9 vectors, HEK293FT cells were seeded in 150 cm^2^ Petri dishes at a density of 5 × 10^4^ cells/cm^2^ and cultured for 48 h to reach ~75–80% confluence. Three hours prior to transfection, the growth medium (DMEM with 4.5 g/L glucose, 10% FBS, penicillin/streptomycin, and non-essential amino acids) was replaced with serum-reduced DMEM.

Cells were co-transfected with three plasmids: (i) pAAV-RC2/9 (AAV2 rep and AAV9 cap), (ii) pHelper, (iii) a transfer plasmid carrying the gene of interest (pAAV-SARS1–5).

Plasmids were mixed in transfection medium at the following doses: target plasmid—0.056 µg/cm^2^, pAAV-RC2/9—0.055 µg/cm^2^, and pHelper—0.089 µg/cm^2^.

Transfection complexes were prepared by separately mixing plasmid DNA and PEI (Polysciences, Warrington, PA, USA) in DMEM, combining the two solutions 1:1 (*v*/*v*), and vortexing for 1 min. The mixture was incubated for 15 min at room temperature and added dropwise to the culture dish containing 20 mL of medium.

After 72 h of incubation at 37 °C and 5% CO_2_, 0.5% Triton X-100 (*v*/*v*) was added directly to the culture. Lysates were collected under sterile conditions, centrifuged at 3000× *g* for 10 min at 4 °C, and the clarified supernatant was harvested. A 50 µL aliquot was retained for viral titer analysis by qPCR, and the remaining lysate was stored at −20 °C.

Viral genome titers were determined using quantitative PCR. Preparations with ≥5 × 10^11^ viral genomes (vg)/mL were considered acceptable for the following applications.

A transfection efficiency control was performed in parallel using pAAV-eGFP instead of the target plasmid. The percentage of GFP-positive cells was analyzed by flow cytometry in FL-1 channel 72 h post-transfection. A transfection efficiency ≥ 90% was considered acceptable.

Recombinant AAV particles were purified by density gradient ultracentrifugation using iodixanol (OptiPrep, Sigma-Aldrich, Saint Louis, MO, USA). Clarified lysates from transfected HEK293FT cells were layered onto pre-formed discontinuous iodixanol gradients prepared in Ultra-Clear™ 16 × 76 mm tubes (Beckman Coulter, Brea, CA, USA) compatible with the 70Ti rotor. The gradients were composed of the following sequential layers: 2 mL of 60% iodixanol (prepared as 58.6% *w*/*v*), 2 mL of 40% iodixanol, 2.75 mL of 25% iodixanol, and 4.5 mL of clarified viral lysate (top layer).

Tubes were centrifuged at 350,000× *g* for 1 h at 18 °C in an Optima XPN or Optima XE ultracentrifuge (Beckman Coulter, Brea, CA, USA). Following ultracentrifugation, the 60% and upper half of the 40% fractions were collected for full capsid preparations. For empty capsid preparations, the upper quarter of the 40% fraction and the top third of the 25% fraction were collected.

Collected fractions were subjected to dialysis using 100 kDa MWCO dialysis tubing (Spectrum Laboratories, Rancho Dominguez, CA, USA, Cat. No. 0867140) against 1× PBS containing 350 mM NaCl and 0.001% Pluronic F-68, at 4 °C with 2–3 buffer exchanges over 14–18 h. The total buffer volume used was at least 200-fold greater than the sample volume.

Samples were then concentrated using centrifugal concentrators with 100 kDa MWCO membranes (Amicon Ultra or equivalent) to a final target concentration of 3 × 10^13^ vg/mL (not applicable to empty capsid preparations). Final products were sterile-filtered using 0.22 µm syringe filters prior to downstream use.

Depending on the expected viral genome titer, samples were assayed at 2 to 6 serial 10-fold dilutions, ensuring that at least two dilutions per sample fell within the dynamic range of the standard curve.

qPCR reactions (25 µL total volume) were prepared with the following components:

• 5 µL of 5× qPCR Master Mix (Syntol, Moscow, Russia),
• 0.025 µL of forward ITR primer (100 µM),
• 0.085 µL of reverse ITR primer (100 µM),
• 0.025 µL of ITR-specific probe labeled with ROX (100 µM),
• 14.865 µL of sterile nuclease-free water,
• 5 µL of sample or pAAV-hrGFP DNA standard.

Negative controls contained sterile water instead of DNA. Reactions were run in triplicate in PCR strip tubes or 96-well plates, sealed, and transferred to a real-time PCR instrument.

Thermal cycling conditions:

Initial polymerase activation: 95 °C for 10 min.

40 cycles of:

• Denaturation: 95 °C for 15 s;
• Annealing/extension: 60 °C for 60 s (fluorescence acquisition);

Melt curve analysis: 55–95 °C with 0.5 °C increments every 15 s.

### 2.5. In Vitro Evaluation of RBD Expression

HEK293FT cells (Thermo Fisher Scientific, Waltham, MA, USA, Cat. No. R70007) were cultured in DMEM/F12 (1:1) supplemented with 10% FBS, 1 mM sodium pyruvate, 1% GlutaMax, and 1% antibiotic–antimycotic (all from Gibco, Waltham, MA, USA). Cells were seeded into 12-well plates (TPP) at a density of 7 × 10^5^ cells per well and incubated at 37 °C with 5% CO_2_ for 24 h until ~30% confluency was reached.

Cells were transduced with 5 × 10^10^ vg per well of recombinant AAV9 vectors carrying SARS-CoV-2 antigen-encoding sequences. Transduction was performed by infection: plates were centrifuged at 2000 rpm for 2 h at 30 °C. Two wells were used per vector construct, and materials were pooled at later steps.

Following transduction, cells were incubated for 6 days under standard culture conditions. At the end of incubation, cells were detached using 0.5% trypsin-EDTA (Sigma-Aldrich, Saint Louis, MO, USA), collected into tubes, and pelleted by centrifugation at 4400 rpm for 5 min. The cell pellets were lysed in 100 µL of Laemmli buffer containing β-mercaptoethanol by boiling in a water bath for 7 min. Lysates were cooled, aliquoted, and stored at −70 °C until analysis.

Expression of the SARS-CoV-2 RBD protein was assessed using dot blot analysis. Briefly, 2 µL of cell lysate was spotted onto a nitrocellulose membrane (Bio-Rad, Hercules, CA, USA) and air-dried at room temperature. Membranes were blocked for 2 h at room temperature on a shaker (250 rpm) in blocking buffer composed of 5% non-fat dry milk (TF Ditol, Saint Petersburg, Russia) in PBS (Biolot, Shchyolkovo, Russia) containing 0.1% Tween-20 (AppliChem, Boca Raton, FL, USA; PBST). Membranes were washed twice with PBST for 10 min.

Primary antibodies against the SARS-CoV-2 RBD—XR-06, XR-25 (Xema Medica, Moscow, Russia), or a 1:1 mixture—were diluted 1:40 in blocking buffer and incubated with the membrane for 2 h at room temperature (250 rpm). After three PBST washes, membranes were incubated for 1 h with Goat Anti-Mouse IgG H&L (HRP-conjugated; Abcam, Cambridge, UK, Cat. No. ab97040), diluted 1:500 in blocking buffer. Following four washes in PBST, membranes were developed using Clarity™ Western ECL substrate (Bio-Rad, Hercules, CA, USA, Cat. No. 1705060) and imaged using the ChemiDoc™ imaging system (Bio-Rad, Hercules, CA, USA).

### 2.6. In Vivo Immunization

Female BALB/c mice were randomly assigned to experimental and control groups (*n* = 7 per experimental group; *n* = 8 for the negative control group; total *n* = 50). Mice in the experimental groups were immunized via intramuscular injection with recombinant AAV9 vectors carrying SARS-CoV-2 antigen sequences (pAAV-SARS2-4; Spike Beta, S1 Beta, and RBD Beta) at doses of either 1 × 10^10^ or 1 × 10^11^ vg per animal. Mice in the negative control group remained untreated (intact).

Blood samples were collected at weeks 4 and 12 post-immunization via submandibular bleeding to assess total anti-viral IgG levels and neutralizing antibody titers.

At week 12, three animals per group were euthanized under approved protocols, and major internal organs—including lungs, liver, kidneys, skeletal muscle, and heart—were harvested for histopathological analysis. Collected tissues were fixed in 10% neutral-buffered formalin for downstream processing.

### 2.7. RT-PCR Analysis

Detection of RNA transcripts encoding target antigens was performed using qualitative reverse transcription PCR (RT-PCR) on tissue homogenates from mice. RNA was extracted from skeletal muscle tissue at the injection site, which served as the primary site of AAV-mediated transgene expression. Total RNA was extracted from 50 µL of tissue suspension using a magnetic bead-based RNA extraction kit (BioLabMix, Novosibirsk, Russia), following the manufacturer’s instructions.

RT-PCR reactions were performed using the BioMaster RT-PCR SYBR Blue (2×) kit (BiolabMix, Novosibirsk, Russia) and primer pair RBD_CO3 fwd (TAC CAC AGA CGC CGT TAG AG) and RBD_CO3 rev (TAC AGC ACT GCC ACC TGA TT) selected from a primer set provided by the customer and validated for optimal amplification efficiency. Amplification and detection were carried out on a CFX96 Real-Time PCR Detection System (Bio-Rad, Hercules, CA, USA).

The thermal cycling profile was as follows:

45 °C—30 min (reverse transcription);

95 °C—5 min (initial denaturation);

95 °C—10 s;

57 °C—10 s;

72 °C—30 s;

82 °C—5 s (fluorescence detection in SYBR channel);

Repeat steps 3–6 for a total of 40 cycles.

### 2.8. Enzyme-Linked Immunosorbent Assay (ELISA)

Serum samples collected from immunized mice were analyzed by ELISA to quantify anti-SARS-CoV-2 or anti-AAV9 IgG antibody titers. The 96-well plates Maxosorp (Nunc) were coated overnight at 4 °C with either inactivated SARS-CoV-2 Beta variant virus (5 µg/mL in PBS; strain hCoV-19/Russia/SPE-RII-27029V3/2021, GISAID: EPI_ISL_8112507) or AAV9 empty capsids (3 × 10^8^ capsids/mL in PBS). Plates were washed twice with PBST (PBS + 0.1% Tween-20) and blocked with 5% non-fat dry milk (TF Ditol, Saint Petersburg, Russia) in PBS for 2 h at room temperature (RT). After blocking, plates were washed twice with PBST.

Serum samples were serially diluted two-fold in PBS, starting from a 1:100 dilution, and added to the wells in duplicate. Plates were incubated for 1 h at RT, followed by three washes with PBST. Goat Anti-Mouse IgG H&L (HRP-conjugated ab97040, Abcam, Cambridge, UK) secondary antibodies were diluted 1:5000 in PBS and added to each well. Plates were incubated for 1 h at RT and washed four times with PBST.

TMB (3,3′,5,5′-Tetramethylbenzidine) substrate (Bioservice, Moscow, Russia) was added and the reaction was developed for 10 min at RT. The enzymatic reaction was stopped with 1N H_2_SO_4_ (Vecton, Saint Petersburg, Russia), and absorbance was measured at 450 nm and 620 nm using a CLARIOstar microplate reader (BMG Labtech, Ortenberg, Germany). The IgG titer was defined as the highest dilution of serum producing an OD (optical density) above the threshold value, which was determined based on the OD of negative control samples and background noise, typically within the range of 0.100–0.200. If the OD at a 1:100 dilution was below the threshold, the titer was recorded as 50.

### 2.9. Virus Neutralization Test (CPE-Based Microneutralization Assay)

The presence of virus-neutralizing antibodies in mouse serum was evaluated using a classical cytopathic effect (CPE)-based microneutralization (MN) assay. Serum samples were heat-inactivated at 56 °C for 30 min and then serially diluted two-fold in AlphaMEM medium (Biolot, Shchyolkovo, Russia) supplemented with 2% FBS (Gibco, Waltham, MA, USA) and 1% antibiotic–antimycotic solution (Gibco, Waltham, MA, USA), starting from a 1:10 dilution (based on undiluted serum).

Diluted serum samples were mixed with an equal volume of SARS-CoV-2 Beta variant virus (strain hCoV-19/Russia/SPE-RII-27029V3/2021, GISAID: EPI_ISL_8112507) at a final concentration of 100 TCID_50_ per well. The virus-serum mixtures were incubated for 1 h at 37 °C and then added to confluent Vero cell monolayers (ATCC, Manassas, VA, USA, Cat. No. CCL-81) seeded at a density of 2 × 10^4^ cells per well in 96-well plates 24 h prior to infection to achieve a confluent monolayer.

Plates were incubated for 4 days at 37 °C in a 5% CO_2_ atmosphere and monitored daily for cytopathic effect. CPE was evaluated visually using an inverted microscope and scored based on cell rounding, detachment, or lysis. The neutralizing antibody titer was defined as the highest dilution of serum that completely prevented visible CPE in all replicate wells. If CPE was observed even at the 1:10 dilution, the neutralizing titer was recorded as 5.

All manipulations with live SARS-CoV-2 were performed in facilities meeting Biosafety Level-2 (BSL-2) requirements for Risk Group-2 pathogens.

### 2.10. Histological Sample Preparation

Organs were fixed in 10% neutral-buffered formalin, placed in histological cassettes, washed under running water for 30 min and processed through a standard series of graded alcohols and paraffin. Paraffin blocks were sectioned at 3–5 µm using a sled microtome (HM430, Thermo Fisher Scientific, Waltham, MA, USA), and sections were mounted onto adhesive glass slides (Biovitrum, Saint Petersburg, Russia). Slides were dried at 37 °C for 12 h, deparaffinized, rehydrated, and stained with hematoxylin and eosin (Biovitrum, Saint Petersburg, Russia). After dehydration and clearing, samples were mounted using a polystyrene-based mounting medium and covered with glass coverslips. Photodocumentation was performed using a Leica DM3000 microscope (Leica, Wetzlar, Germany) connected to an IBM PC and operated with LAS X software 5.0.2 (Leica, Wetzlar, Germany).

### 2.11. Immunofluorescence Staining

Deparaffinized tissue sections underwent antigen retrieval in Trilogy buffer (Sigma-Aldrich, Saint Louis, MO, USA), followed by three washes in PBS (pH 7.4; Nevareaktiv, Saint Petersburg, Russia). Slides were treated with Background Block solution (Cell Marque, Rocklin, CA, USA) and incubated for 1 h in a humidified chamber. After removing excess blocking solution, sections were incubated for 2 h at room temperature with primary rabbit monoclonal antibodies against the SARS-CoV-2 Spike protein (GTX135356, GeneTex, Irvine, CA, USA), diluted 1:1000 in Diamond antibody diluent (Cell Marque, Rocklin, CA, USA, Cat. No. 938B-05).

Following three PBS washes, sections were incubated for 1 h at room temperature with Alexa Fluor 555-conjugated goat anti-rabbit IgG secondary antibodies (Abcam, Cambridge, UK, Cat. No. ab150078; 1:500 dilution). Slides were then washed, counterstained with DAPI (Thermo Fisher Scientific, Waltham, MA, USA, Cat. No. D1306) for 10 min, washed again with distilled water, and mounted in Kaiser’s glycerogel mounting medium under coverslips.

Samples were analyzed using a Zeiss LSM 5 Pascal confocal (laser scanning) microscope (Carl Zeiss, Oberkochen, Germany). Fluorescence channels were separated using filter sets for DAPI (420 nm), autofluorescence (515–530 nm), and Alexa Fluor 555 (565–590 nm). Red fluorescence localized in the cytoplasm and membrane was interpreted as a positive signal. Lung tissue from COVID-19 autopsy cases served as positive controls.

### 2.12. Isolation, Stimulation, and Flow Cytometry Analysis of Splenocytes

Murine spleens were aseptically harvested into RPMI-1640 medium (Biolot, Shchyolkovo, Russia) supplemented with penicillin/streptomycin (P/S) (PanEko, Moscow, Russia) and homogenized. Cell suspensions were filtered through 70 µm cell strainers (PluriSelect, Leipzig, Germany), washed in DPBS (Biolot, Shchyolkovo, Russia) containing 2% heat-inactivated (HI) FBS (Gibco, Waltham, MA, USA) and P/S, and centrifuged at 500× *g* for 7 min at 12 °C. Red blood cells were lysed using RBC lysis buffer (Thermo Fisher Scientific, Waltham, MA, USA), followed by a second wash. Cell viability was assessed using the Zombie Aqua viability dye (BioLegend, San Diego, CA, USA, Cat. No. 423101) on a CytoFLEX flow cytometer (Beckman Coulter, Brea, CA, USA). Flow cytometry reagent list is presented in Table S3.

Viable splenocytes were resuspended in RPMI-1640 medium supplemented with 10% HI FBS and 1% P/S and plated in flat-bottom 96-well plates (TPP, Trasadingen, Switzerland) at 1 × 10^6^ cells/100 µL per well. Cells were stimulated for 24 h at 37 °C and 5% CO_2_ with PepTivator^®^ SARS-CoV-2 Prot_S (Miltenyi Biotec, Bergisch Gladbach, Germany), an overlapping peptide pool covering the full Spike glycoprotein sequence (GenBank: MN908947.3). The culture medium also contained brefeldin A (BioLegend, San Diego, CA, USA) and anti-CD28 costimulatory antibodies (BioLegend, San Diego, CA, USA, Cat. No. 102105). Unstimulated cells (brefeldin A and anti-CD28 only) served as negative controls, and a mixture of PMA (Sigma-Aldrich, Saint Louis, MO, USA, Cat. No. 50-165-6915) and ionomycin (Sigma-Aldrich, Saint Louis, MO, USA, Cat. No. I0634) was used as a positive stimulation control.

Following stimulation, cells were fixed, permeabilized, and stained using the CytoFix/CytoPerm intracellular staining kit (BD Biosciences, San Jose, CA, USA, Cat. No. 554714) according to the manufacturer’s protocol. Cells were labeled with fluorochrome-conjugated antibodies against CD4 (PC5.5; BD Biosciences, San Jose, CA, USA, Cat. No. 332772), CD8 (PC7; BioLegend, San Diego, CA, USA, Cat. No. 344713), CD62L (APC-A750; BioLegend, San Diego, CA, USA, Cat. No. 104449), CD44 (KO525; BioLegend, San Diego, CA, USA, Cat. No. 103011), IFNγ (FITC; BioLegend, San Diego, CA, USA, Cat. No. 506504), TNFα (PB450; BioLegend, San Diego, CA, USA, Cat. No. 430904), and IL-2 (PE; BioLegend, San Diego, CA, USA, Cat. No. 431807). The viability marker Zombie Aqua was included to exclude dead cells, and TruStain FcX (anti-mouse CD16/32; BioLegend, San Diego, CA, USA, Cat. No. 101319) was used to block non-specific Fc receptor binding.

Data were acquired on a CytoFLEX flow cytometer and analyzed using Kaluza Analysis 2.1 software. The gating strategy involved an exclusion of dead cells based on FSC/SSC (Forward Scatter/Side Scatter) and viability staining, identification of CD4^+^ helper and CD8^+^ cytotoxic T cells, and classification of memory subsets (central memory: CD44^+^CD62L^+^; effector memory: CD44^+^CD62L^−^). Cytokine-producing effector memory T cells were identified based on expression of IFNγ, TNFα, and IL-2.

### 2.13. Primary Data and Statistical Processing

Primary data were processed in Microsoft Excel and exported to GraphPad Prism v8.4.3 (GraphPad Software, Boston, MA, USA) for visualization and statistical analysis. To assess the dynamics of antibody production, geometric mean titers (GMT) were calculated for each group at specified time points post-immunization. Antibody titers were log-transformed prior to statistical analysis to ensure normal distribution.

Group comparisons were performed using one-way ANOVA followed by Tukey’s post hoc test for normally distributed data, and the nonparametric Kruskal–Wallis test followed by Dunn’s multiple comparison test for antibody titers. A *p*-value of <0.05 was considered statistically significant. Raw data tables and detailed statistical outputs are provided in Supplementary Information.

## 3. Results

### 3.1. Design of SARS-CoV-2 Spike Protein Antigen Constructs

Five antigen-encoding constructs were designed based on the Spike (S) protein of SARS-CoV-2, each cloned into the pAAV-CMV expression vector for subsequent AAV packaging. The modifications for each construct are summarized below:

• Construct 1—Full-length Spike (Wuhan variant). It encodes the full-length S protein (residues 1–1273) with the following stabilizing mutations: deletion of the furin cleavage site (RRAR682A), double proline substitution at positions 986–987 (K986P, V987P), and D614G substitution. This construct does not contain additional variant-specific mutations and serves as a reference wild-type backbone.
• Construct 2—Full-length Spike (Beta variant). Same as Construct 1, but additionally contains additional RBD mutations characteristic of the Beta (B.1.351) variant: K417N, E484K.
• Construct 3—S1 Subunit (Beta variant). It encodes the S1 subunit (residues 1–680) of the Spike protein, including: D614G substitution, Beta-specific mutations K417N and E484K, and a stop codon after residue 680.
• Construct 4—RBD (Beta variant). It encodes only the receptor-binding domain (RBD), including: RBD flanked by upstream and downstream Spike regions for proper folding (residues ~302–684), D614G, K417N, and E484K mutations.
• Construct 5—Full-length Spike (Delta+ variant). It encodes the full-length S protein with mutations characteristic of the Delta+ lineage, including: L452R and T478K mutations in the RBD, D614G, RRAR682A deletion, and K986P-V987P stabilizing substitutions.

The full-length Spike was engineered to include several stabilizing and immunologically relevant mutations. The RRAR682A deletion eliminates the polybasic furin cleavage site between S1 and S2, preventing premature cleavage and enhancing antigen stability. The K986P-V987P double substitution (commonly used in prefusion-stabilized Spike constructs) enhances conformational stability of the trimer, preserving native epitopes recognized by neutralizing antibodies [27]. The D614G mutation, which became globally dominant early in the pandemic, improves Spike trimer stability and viral infectivity, and is associated with enhanced immunogenicity [28]. The main K417N and E484K mutations, characteristic of the Beta variant, were included to represent immune-evasive features of this variant [29].

Among the five designed plasmids, three representative Beta-variant constructs—full-length Spike, the S1 subunit, and the RBD—were selected for in vivo immunization studies, as they reflect the main antigen formats for comparative analysis. The remaining two variant constructs (Wuhan and Delta+) were used to validate cloning efficiency and confirm in vitro expression only.

**Figure 1:**
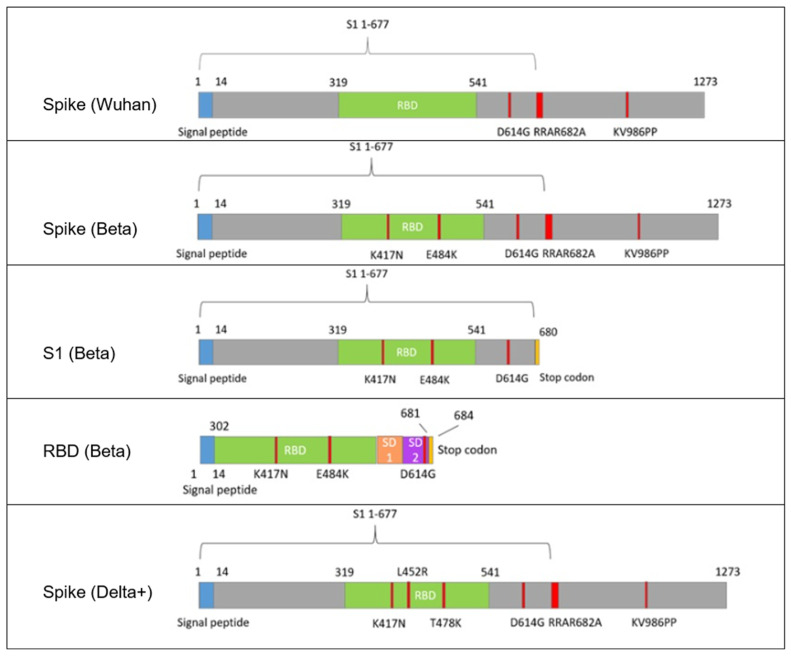
Schematic representation of SARS-CoV-2 Spike-based antigen constructs used in AAV9 vaccine development. (1) Spike (Wuhan)—full-length Spike (1–1273) with RRAR682A (furin site deletion), K986P/V987P (prefusion stabilization) and D614G substitutions. (2) Spike (Beta)—same as (1) but additionally carrying RBD mutations K417N and E484K. (3) S1 (Beta)—S1 subunit (1–680) with D614G, K417N, E484K; lacks S2 and transmembrane regions. (4) RBD (Beta)—RBD (302–684) flanked by SD1/SD2 regions, including K417N, E484K, D614G. (5) Spike (Delta+)—full-length Spike with L452R, T478K, D614G, RRAR682A deletion, and K986P/V987P stabilizing substitutions. Color legend: green–RBD part; blue–signal peptide; gray–the remaining part of the Spike protein; red–individual point mutations; orange and purple–SD1 and SD2 domains, respectively. All constructs were codon-optimized for mammalian expression under the CMV promoter and packaged into AAV9 vectors for comparative immunogenicity analysis.

All constructs were validated by restriction digestion and Sanger sequencing. Figure 1 illustrates schematic representations of each construct and key mutations. Each construct encodes a different version of the Spike protein or its subdomains, codon-optimized for mammalian expression. Constructs include: (1) wild-type Wuhan full-length Spike with modifications (RRAR682A, K986P-V987P, D614G); (2) full-length Spike with Beta variant RBD mutations (K417N, E484K); (3) S1 subunit with Beta-RBD mutations; (4) RBD flanked by SD1 and SD2 (Subdomain 1 and Subdomain 2) with Beta-specific mutations; (5) full-length Spike with Delta+ mutations (L452R, T478K). All constructs include D614G. Key mutations and domain boundaries are annotated.

### 3.2. In Vitro and In Vivo Expression of SARS-CoV-2 Antigens

**Figure 2:**
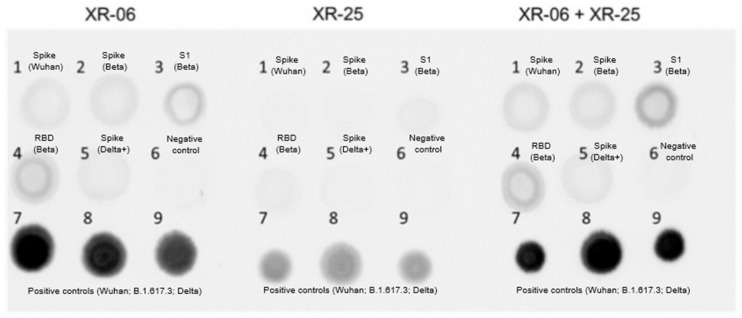
Dot blot analysis showed cell lysates that were applied to the membrane, which were then stained with monoclonal antibodies to RBD or a mixture of them (XR-06, XR-25). Sample numbers: (1) Spike (Wuhan); (2) Spike (Beta); (3) S1 (Beta); (4) RBD (Beta); (5) Spike (Delta+); (6) negative control, non-transduced cells; (7) positive control, cells infected with the Wuhan B1 line; (8) positive control, Delta-related lineage B.1.617.3; (9) positive control, Russian Delta line AY.122.

Dot blot analysis confirmed that all tested recombinant AAV9 vectors (Spike (Wuhan), Spike (Beta); S1 (Beta); RBD (Beta); Spike (Delta+); rAAV9-SARS1-5) successfully expressed the RBD region of the Spike protein. However, the expression levels varied substantially between constructs. The highest antigen expression was observed in cells transduced with rAAV9-SARS4 (RBD Beta) and rAAV9-SARS3 (S1 Beta ), while all full-length Spike constructs (rAAV9-SARS1,2,5) demonstrated a markedly lower signal intensity. These differences may reflect differences in transcriptional or translational efficiency depending on insert size and structure (Figure 2).

Interestingly, despite the lower in vitro signal intensity of the full-length Spike construct compared with S1 and RBD in dot-blot analysis, this vector induced the strongest humoral and cellular immune responses in vivo. This inverse relationship suggests that antigen structure and membrane anchoring, rather than expression level alone, play a dominant role in shaping the immunogenicity of AAV-delivered antigens.

Immunofluorescence staining was performed for all AAV9-based constructs. Specific Spike signal was detected only in muscle sections from mice immunized with the full-length Spike vector, while no detectable signal was observed for the S1 or RBD constructs. The absence of staining for these truncated antigens may reflect their faster intracellular degradation and lack of membrane anchoring, which can reduce persistence in fixed tissue. Nevertheless, mice immunized with S1 and RBD demonstrated detectable antigen-specific IgG responses, indicating that in vivo antigen expression did occur, albeit transiently or at levels below the detection threshold of the assay.

**Figure 3:**
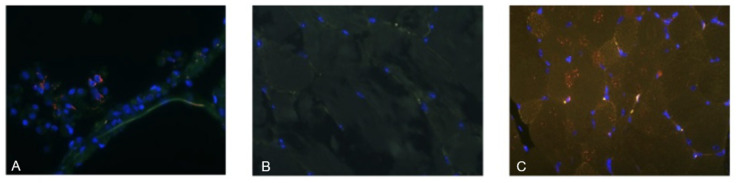
Immunofluorescence detection of SARS-CoV-2 Spike protein in murine tissue. Representative sections of skeletal muscle or lung tissue were stained with anti-Spike monoclonal antibody (EpigenTek, SARS-CoV-2 Spike Monoclonal Antibody). (**A**) Positive control—lung tissue from a confirmed SARS-CoV-2 case (autopsy sample). (**B**) Negative control—skeletal muscle from an untreated mouse. (**C**) Experimental group—skeletal muscle from a vaccinated mouse, 4 weeks post-administration of the vaccine, showing specific Spike protein expression at the injection site. Fluorescence: red—SARS-CoV-2 Spike protein, green—tissue autofluorescence, blue—DAPI nuclear stain. Magnification: ×100.

Immunofluorescence staining revealed the presence of SARS-CoV-2 Spike protein in tissue sections from mice immunized with AAV-based vaccine constructs. Specific red fluorescence, corresponding to Alexa Fluor 555–labeled anti-Spike antibodies, was observed in skeletal muscle at the injection site (Figure 3C). Comparable staining was also detected in the positive control tissue (lung samples from COVID-19 patients), confirming antibody specificity and detection accuracy (Figure 3A). No specific signal was observed in tissues from the negative control group, supporting the specificity of vector-mediated antigen expression (Figure 3B). These findings provide direct evidence of successful in vivo expression of the Spike protein following AAV9-mediated gene delivery and validate the use of immunofluorescence as a complementary approach to dot blot and serological assays.

### 3.3. Evaluation of the Humoral Immune Response to SARS-CoV-2 Antigens Delivered via AAV9-Based Vaccine in a Murine In Vivo Model

The neutralization assay in this study was performed using the Beta variant, as all three AAV constructs encoded Beta Spike–derived antigens. This design allowed us to directly compare antigen formats under matched antigenic conditions. Mice were immunized intramuscularly with adeno-associated vectors according to the study design. Groups 1 and 2 were administered the variant encoding the full-length Spike protein at doses of 10^10^ vg and 10^11^ vg, respectively. Groups 3 and 4 were administered the variant encoding the S1 subunit at doses of 10^10^ vg and 10^11^ vg, respectively. Groups 5 and 6 were administered the variant encoding the RBD at doses of 10^10^ vg and 10^11^ vg, respectively. Group 7 consisted of naive animals. For 2 weeks after immunization, the weight of mice fluctuated within ±5% of the initial weight. The exception was a short-term weight loss in 3/7 mice in group 6 (RBD, dose 10^10^), observed on days 13–14 (data not attached).

**Figure 4:**
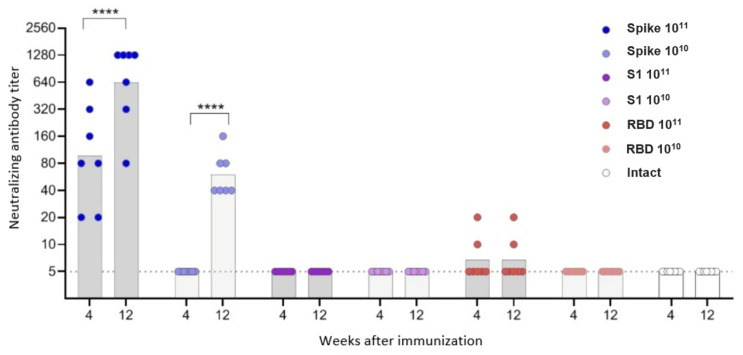
Neutralizing antibodies to the Beta variant of the SARS-CoV-2 in the sera of immunized mice. The dots represent individual values for each animal; the columns represent the geometric mean titers (GMT) by groups. **** *p* < 0.0001, statistically significant difference in titers between 4 and 12 weeks (ANOVA with Tukey’s post hoc; Kruskal–Wallis test with Dunn’s multiple comparisons).

The study of the antibody response in vaccinated animals showed that virus-neutralizing antibodies were formed only in mice vaccinated with the Spike construct at both doses and in some mice (2/7) vaccinated with the RBD construct at a high dose. Antibody titers in mice vaccinated with the Spike construct significantly increased by the 12th week after immunization and had a direct dose dependence (Figure 4).

**Figure 5:**
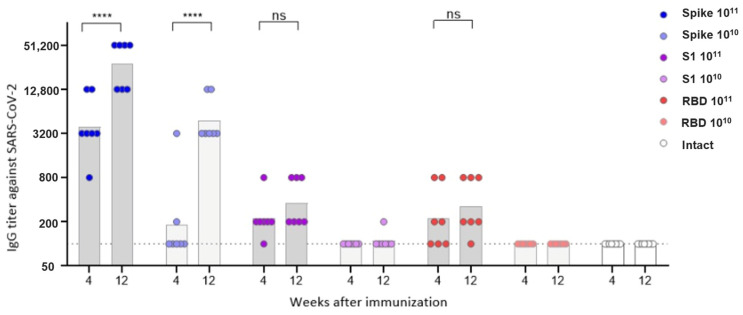
IgG antibodies to the Beta variant of the SARS-CoV-2 in the sera of immunized mice. The dots represent individual values for each animal; the columns represent geometric mean titers (GMT) for each group. **** *p* < 0.0001, statistically significant difference in titers between 4 and 12 weeks; ns—not significant (ANOVA with Tukey’s post hoc test; Kruskal–Wallis with Dunn’s multiple comparisons for nonparametric data).

In contrast to neutralizing antibodies, virus-specific IgG antibodies were observed in animals vaccinated with all three constructs, but for the S1 and RBD vectors—only at a high dose (Figure 5, Table S4). Antibody titers had a direct dependence on the insert length: the highest antibody level was observed in mice vaccinated with Spike, the lowest—in mice vaccinated with RBD. Antibody titers increased in the period of 4–12 weeks, the difference was statistically significant for the Spike construct.

**Figure 6:**
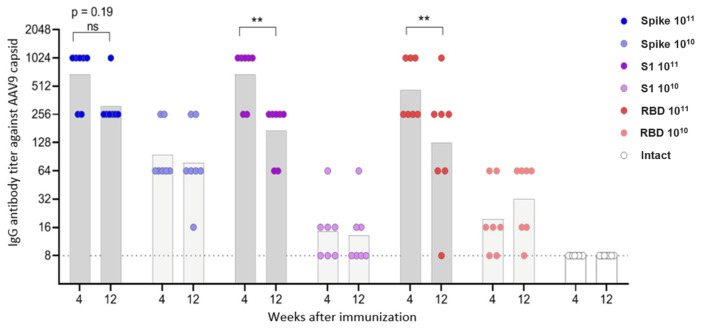
IgG antibodies to AAV9 capsid protein in sera of immunized mice. The dots represent individual values for each animal; the columns represent geometric mean titers (GMT) by groups. ** *p* < 0.01, statistically significant difference in titers between 4 and 12 weeks; ns—not significant (ANOVA with Tukey’s post hoc; Kruskal–Wallis with Dunn’s multiple comparisons for nonparametric data).

The humoral response to the AAV9 capsid antigen 4 weeks after vaccination was directly dose-dependent, with no differences between the different vector groups (Figure 6, Table S4). At 12 weeks after immunization, a statistically significant decrease in AAV9 antibodies was observed in the groups of mice vaccinated with the high dose of the S1 and RBD constructs. In the group of mice vaccinated with the high dose of the Spike construct, a decrease in antibody titer was also observed, but it was not statistically significant. In the other groups, AAV9 antibody titers remained virtually unchanged over the period of 4–12 weeks.

### 3.4. Study of T-Cell Immune Response of Experimental Proteins in the Context of an AAV9-Based Vaccine During In Vivo Immunization of Mice

**Figure 7:**
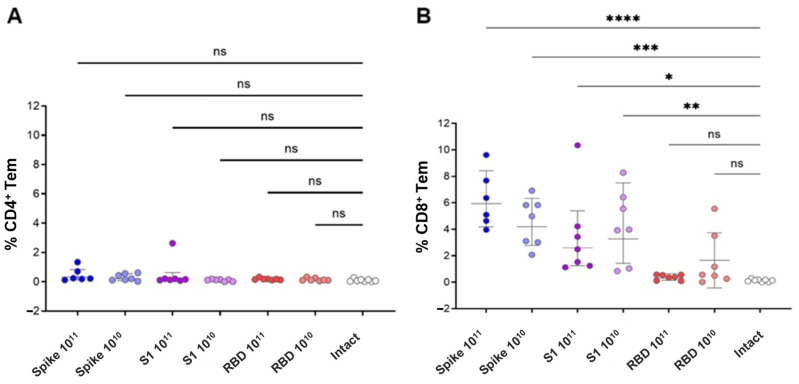
Cumulative antigen-specific cytokine production by effector CD4^+^ and effector CD8^+^ memory T cells in response to immunization with the studied drugs. The graphs show individual values of the cumulative proportion of cytokine-producing T lymphocytes from the total number of effector CD4^+^ (**A**) and CD8^+^ (**B**) T cells (CD4/8^+^/CD44^+^/CD62L^−^) upon stimulation with PepTivator S. Groups were compared using one-way ANOVA with subsequent comparison of the study groups with the group of naive animals using Dunnett’s test, * *p* < 0.05, ** *p* < 0.01; *** *p* < 0.001, **** *p* < 0.0001, ns (non-significant) *p* > 0.05.

Systemic antigen-specific T-cell response was determined 12 weeks after vaccination. Spleens were collected from all animals, followed by isolation of splenocytes. The response was assessed by the relative number of IFNγ/IL-2/TNFα cytokine-producing CD4^+^ and CD8^+^ effector memory T-cells, measured by flow cytometry, after in vitro stimulation of splenocytes with a cocktail of overlapping S-protein peptides (PepTivator S). Immunization with the studied drugs induced a predominantly CD8^+^ effector T-cell response, the intensity of which was determined by the size of the insert expressed by the adeno-associated vector (Figure 7A,B).

Statistical analysis for Figure 7 was performed using one-way ANOVA with Dunnett’s post-test versus naïve controls. Therefore, the significance indicators in the figure reflect comparisons with naïve animals only. While the numerical differences among vaccine constructs were small (1–3%), this pattern was consistent across biological replicates and reproducible across multiple cytokines (IFNγ, IL-2, TNFα). The full-length Spike construct displayed the highest mean frequencies descriptively, supporting its relatively stronger immunogenic potential in vivo.

**Figure 8:**
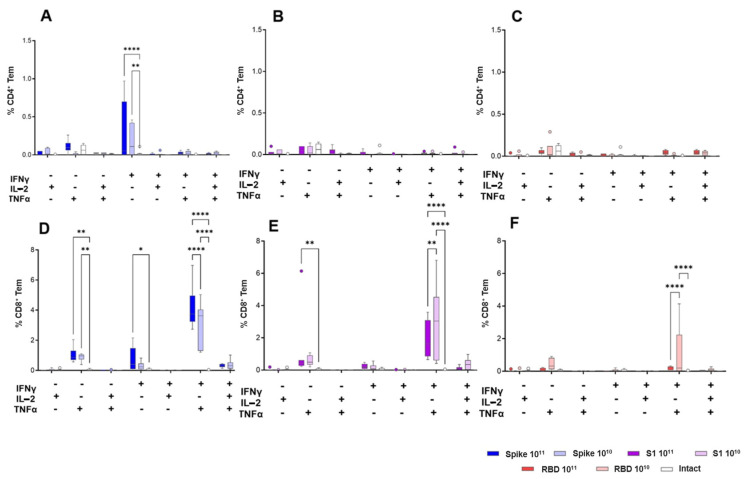
Subpopulation composition of cytokine-producing CD4^+^ (**A**–**C**) and CD8^+^ (**D**–**F**) Tem in mouse spleens in response to immunization with the studied drugs. The graphs show the data reflecting the percentage of different subpopulations of cytokine-producing CD4^+^ and CD8^+^ cells in the total CD4/8^+^/CD44^+^/CD62L^−^ Tem population. Groups were compared using ANOVA with subsequent pairwise comparison using the Tukey test. * *p* < 0.05, ** *p* < 0.01, **** *p* < 0.0001.

The main proportion of antigen-specific effector CD8^+^ T lymphocytes was made up of the population of dual (IFNγ+/IL-2-/TNFα+) producers. The CD4^+^ antigen-specific T cell response was observed only in the group immunized with the adeno-associated vector expressing the full-length Spike protein and was characterized by the predominance of monofunctional IFNγ+/IL-2-/TNFα- Tem cells (Figure 8).

Analysis of the total cytokine production (IFNγ/IL-2/TNFα) did not reveal a pronounced dose-dependent effect for all the studied constructs. Differences were noted when analyzing the proportion of IFNγ+/IL-2-/TNFα+ CD8^+^ Tem lymphocytes. In the groups immunized with the adeno-associated vector expressing the full-length Spike protein, a direct relationship was observed between the dose and the relative number of IFNγ+/IL-2-/TNFα+ CD8^+^ Tem cells, and in the groups immunized with the S1 and RBD constructs, an inverse relationship was observed. The differences observed here were statistically significant (*p* < 0.05, one-way ANOVA with Tukey’s test) and consistent with the overall pattern of stronger responses induced by the full-length Spike construct across humoral and cellular assays.

### 3.5. Histological Analysis

Histochemical examination of the internal organs and vaccine injection site (skeletal muscles) was performed on the material represented by the tissue cross-section. Histological analysis of skeletal muscle tissue from animals in the experimental group revealed transient local changes consistent with a mild inflammatory response to AAV9-based vaccination.

**Figure 9:**
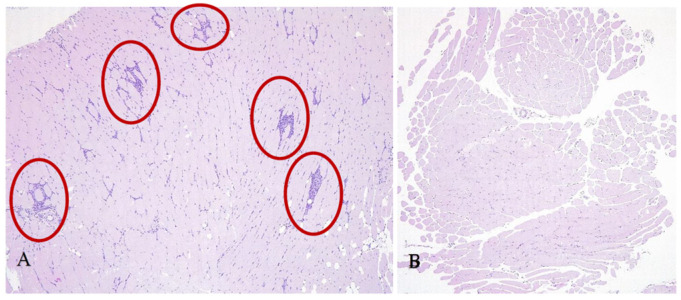
Representative cross-sections of skeletal muscle from mice 4 weeks after AAV9-based vaccine administration. Injection-site muscle showing mild endomysial inflammatory infiltration (red circles) (**A**). Intact muscle tissue from a control animal with no pathological changes (**B**). Staining: hematoxylin and eosin; magnification ×100.

In the early phase of the study (week 4), several animals exhibited multiple foci of endomysial inflammation. These lesions were characterized by clusters of histiocytes and lymphocytes surrounding viable muscle fibers with internalized nuclei—typical features of early post-injury inflammatory response (Figure 9A). Affected muscles also displayed a pronounced regenerative pattern, including a high proportion of myofibers with centrally located nuclei (up to 70–90%), rounded fiber morphology, and signs of endomysial lipomatosis, reflecting tissue remodeling after transient damage.

Importantly, the severity and frequency of these changes declined over time. By week 12, most lesions had regressed, and by week 24, no foci of inflammation or structural abnormalities were observed. In some animal muscles both reactive lesions and intact regions were found during intermediate time points, whereas others exhibited no detectable pathology. These findings suggest that the observed local changes were self-limiting and not associated with systemic dissemination of the viral vector.

No inflammatory changes were observed in skeletal muscles of mice injected with the control vector or in untreated animals (Figure 9B), supporting the conclusion that local reactive changes were directly associated with antigen expression induced by the AAV9-based vaccine. Importantly, these changes were transient in nature, progressively regressing over time and fully resolving by week 24 post-immunization.

**Figure 10:**
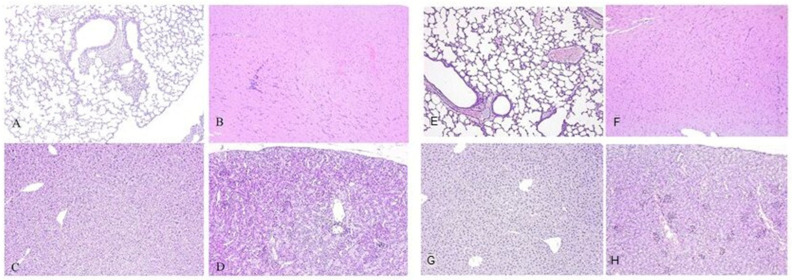
Representative cross-sections of lung (**A**), heart (**B**), liver (**C**), kidney (**D**) of mice from the experimental group, 12 weeks after the introduction of the viral vector; and cross-sections of lung (**E**), heart (**F**), liver (**G**), kidney (**H**) of mice from the control group. Staining: hematoxylin and eosin. Magnification ×100.

No pathological changes were observed in the lungs, heart, liver, or kidneys in any of the animal groups throughout the entire study period and in all groups (Figure 10A–H).

No signs of local tissue damage or inflammation were observed in mice that received the control viral vector or in animals from the intact (non-immunized) group, suggesting that the observed reactive changes were specifically associated with the administration of the antigen-encoding AAV9 vector. The inflammatory response was localized strictly to the injection site in the skeletal muscle and did not extend to adjacent muscle groups. No pathological alterations were detected in internal organs across any of the experimental or control groups, indicating the absence of systemic dissemination of the viral vector and supporting the overall safety profile of the vaccine. These findings suggest that the vector-based vaccine induces local, transient immunogen expression sufficient to trigger a localized immune response, without causing systemic toxicity or long-term tissue damage.

## 4. Discussion

In this study, we investigated the immunogenicity of recombinant AAV9 vectors encoding different fragments of the SARS-CoV-2 Spike protein: the full-length prefusion-stabilized Spike, the S1 subunit, and RBD. Despite comparable delivery routes, doses, and in vitro expression confirmed by dot blot analysis, only the full-length Spike construct induced a detectable humoral response in vivo. Notably, the construct showing the lowest in vitro expression signal (full-length Spike) produced the highest antibody and T-cell responses, whereas constructs with higher expression (S1 and RBD) were weakly immunogenic. This observation supports the view that correct antigen conformation and surface presentation, not absolute expression level, determine immunogenicity in the AAV context. Mice immunized with either the S1 or RBD constructs failed to develop measurable anti-Spike or neutralizing antibodies. A higher dose of the drug and a longer antigen insert were associated with a higher level of both humoral and T-cell responses to the S protein of the SARS-CoV-2 virus.

Neutralizing antibodies were detected only in animals vaccinated with the Spike construct; the antibody titer increased in the period 4–12 weeks after vaccination and had a direct dose-dependent effect. Virus-specific IgG antibodies were observed in animals vaccinated with all three constructs, but for the S1 and RBD vectors, only at a high dose. IgG antibody titers were directly dependent on the insert length and increased over the period of 4–12 weeks.

Immunization of animals resulted in the formation of a pronounced CD8^+^ T-cell response to the peptides of the S protein of the SARS-CoV-2 virus, the intensity of which did not have a clear dose dependence, but directly depended on the insert size. In the population of antigen-specific CD8^+^ effector memory cells, double-positive cells (IFNγ+/TNFα+) predominated. An antigen-specific CD4^+^ T-cell response was recorded only in mice vaccinated with the Spike construct, with IFNγ-producing cells predominating in the population.

The choice of antigen format is a critical determinant of immune outcomes in AAV-based vaccine platforms. While RBD- and S1-based immunogens have demonstrated high efficacy in protein subunit vaccines, where multimerization and adjuvants compensate for their small size, genetic vaccine platforms such as AAV, mRNA, or DNA often require larger or membrane-anchored antigens to elicit robust immunity. Full-length Spike, presented in its native conformation and orientation on the host cell surface, supports efficient B-cell receptor engagement and T-cell priming via MHC presentation. In contrast, smaller soluble antigens such as monomeric RBD may be less efficiently processed, more rapidly degraded, or inadequately trafficked to lymphoid tissues when expressed in vivo.

Multiple groups have developed AAV-based vaccines against SARS-CoV-2, exploring a range of antigen formats and serotypes to optimize immunogenicity and durability [30,31,32]. Full-length or large Spike-derived constructs have shown consistently strong performance. The AC1 candidate, expressing a prefusion-stabilized full-length Spike, and AC3, encoding a secreted S1 subunit, both based on AAVrh32.33, elicited high, long-lasting humoral and cellular immune responses in macaques, protecting against lung pathology following viral challenge [25]. Similarly, an AAV5-based platform encoding full Spike induced durable neutralizing titers. In a head-to-head comparison of multiple Spike-derived formats within the same AAV5 platform, the RBD-plus construct (RBD extended by 42 additional amino acids) elicited the highest IgG titers and neutralizing antibody responses at all measured time points, while the monomeric RBD generated intermediate responses, and S1 failed to induce detectable neutralizing antibodies. These results indicate that either extending the RBD with additional residues or presenting the full Spike structure is advantageous for eliciting strong humoral immunity in an AAV context [33].

Notably, RBD-based constructs have also demonstrated strong immunogenicity when structurally optimized or multimerized. The AAV9-RBD vaccine encoded two copies of the RBD, separated by a linker, resulting in efficient secretion and high neutralizing antibody titers in mice eight weeks after intramuscular or intranasal administration, accompanied by elevated levels of IFNγ, IL-2, IL-4, and IL-10. The S663V-RBD candidate, based on AAV6 and encoding a trimeric RBD fused to the RS09 adjuvant, induced rapid and long-lasting RBD-specific IgG responses in mice, superior neutralizing activity against wild-type, Lambda, and Delta pseudoviruses compared to the licensed BBIBP-CorV vaccine, and overcame pre-existing anti-capsid immunity [30]. A series of multimeric RBD constructs, AAV-RBD(max), AAV-RBD(wt), AAV-2xRBD, and AAV-3xRBD, produced potent, durable immune responses in mice for at least six months, with the 3×RBD format achieving the highest neutralizing activity and also eliciting strong responses in dogs [34]. Furthermore, an AAV2/9 vector expressing a thermostabilized SRBD (with Q321–S591 β-sheet region) elicited complete seroconversion at medium and high doses in macaques, with antibody titers sustained for up to 598 days and cross-neutralization of Beta, Delta, and Omicron variants, without signs of toxicity [35].

Collectively, these findings indicate that for AAV gene delivery, unmodified monomeric RBD is generally suboptimal. Robust performance is achieved when the RBD is multimerized, structurally stabilized, fused to an adjuvant, or extended with additional sequences. Full-length Spike remains a reliable immunogen for eliciting broad and durable immunity; however, optimized RBD-based designs might, in some models, match or even surpass full-length antigens while offering advantages in genetic payload constraints and potential manufacturing efficiency.

To date, no adenoviral COVID-19 vaccine has been deployed clinically using only the RBD or S1 subunit; all authorized adenoviral vector vaccines, including ChAdOx1-nCoV-19 (AstraZeneca/Oxford), Ad26.COV2.S (Johnson & Johnson) and Gam-COVID-Vac (Sputnik V) encode the full-length SARS-CoV-2 spike protein [9,36,37]. This consistent design choice underscores that antigen structure and multimerization are critical for immune visibility, particularly in gene-delivery platforms where no external adjuvant is present.

Mechanistically, larger or membrane-anchored antigens may persist longer at the injection site, be more effectively presented via MHC class I and II pathways, and display native conformational epitopes that efficiently engage B cells. In contrast, small soluble proteins such as monomeric RBD may diffuse rapidly and lack sufficient immunostimulatory context, limiting both B-cell activation and T-cell help. This principle is consistent with findings in the DNA vaccine platform, where trimerized and ferritin-fused HA (hemagglutinin) vaccines elicited significantly stronger humoral and cellular immunity and better protective efficacy compared to conventional (monomeric) HA constructs [38].

Taken together, our results support the conclusion that full-length Spike is generally the optimal immunogen for AAV-based vaccines, likely due to its structural integrity, membrane anchoring, and broader repertoire of B- and T-cell epitopes. This contrasts with protein-based platforms, where shorter antigens such as RBD can be highly immunogenic if multimerized and paired with potent adjuvants. Overall, antigen selection for genetic vaccines must consider not only epitope content but also structural context, degree of multimerization, and in vivo presentation to ensure optimal immunogenicity.

### Limitations

This study has several limitations. First, neutralizing activity was evaluated using only a single SARS-CoV-2 isolate (Beta variant), because all AAV constructs encoded Beta-derived Spike antigens. While this design allowed a controlled comparison of antigen formats, it does not capture potential differences in cross-variant breadth. Second, neutralization was not assessed against currently circulating Omicron-lineage strains (such as XBB or JN.1), which exhibit substantial antigenic divergence from the Beta variant.

Third, antigen expression was assessed qualitatively by dot blot and immunofluorescence, without quantitative measurements of protein abundance or secretion dynamics. Fourth, the kinetics of in vivo antigen expression were not evaluated; therefore, possible differences in expression persistence between full-length and truncated antigens remain uncharacterized.

Fifth, the mechanistic basis for the superior immunogenicity of the full-length Spike—despite lower in vitro signal intensity—remains to be fully elucidated. Future studies incorporating antigen trafficking, conformational analyses, or APC (antigen-presenting cells)-targeting experiments may help clarify these observations. Finally, only two vector doses were tested, preventing full characterization of dose–response relationships, particularly for truncated antigens.

Future work addressing these limitations will help define optimal antigen formats for AAV-based vaccine design and clarify the mechanistic drivers of antigen-specific immunity in this platform.

## 5. Conclusions

In this head-to-head evaluation of AAV9-delivered SARS-CoV-2 antigens, the prefusion-stabilized full-length Spike consistently outperformed truncated formats, inducing robust, dose-responsive neutralizing antibodies and the strongest CD8^+^ effector-memory responses, while uniquely eliciting a measurable CD4^+^ T-cell response; by contrast, S1 failed to generate neutralization and RBD produced neutralizing activity only in a subset of high-dose recipients, despite detectable binding IgG. Antigen-specific IgG scaled with insert length (Spike > S1 > RBD), and safety profiling revealed only transient, localized myositis without systemic pathology, consistent with on-target antigen expression at the injection site. Mechanistically, these data support the principle that, in gene-delivery contexts, larger or membrane-anchored antigens better preserve native conformational epitopes and enhance antigen processing/presentation, whereas small soluble domains require structural optimization (e.g., multimerization, stabilization, or adjuvant fusions) to approach comparable potency. Collectively, our findings position full-length Spike as the preferred immunogen for AAV-based COVID-19 vaccines and provide a clear design framework for next-generation constructs; either maintain full-length Spike or engineer compact RBD formats that explicitly incorporate multimerization/stabilization to overcome the intrinsic limitations of short, soluble antigens.

## Data Availability

The datasets generated or analyzed during this study are available from the corresponding author on a reasonable request.

## Supplementary Materials

The following supporting information can be downloaded at: https://www.mdpi.com/article/10.3390/vaccines13121187/s1, Table S1: The following oligonucleotide primers were used for ligation-independent cloning and colony PCR to generate the SARS-CoV-2 antigen-expressing constructs; Table S2: Primer Sequences for Sanger Sequencing; Table S3: List of antibodies and reagents used in flow cytometry; Table S4. Geometric Mean Titers of antibodies in immunized animal groups. Figure S1: Validation of plasmid constructs by restriction analysis. Maps of the pAAV-SARS1-5 plasmids, theoretical restriction digest schemes, and corresponding agarose gel electrophoresis results. Plasmids were digested with specific restriction enzymes as indicated: pAAV-SARS1—NheI/BbsI; pAAV-SARS2 and pAAV-SARS3—MluI; pAAV-SARS4—BstBI/MluI; pAAV-SARS5—EcoRV. The observed fragment sizes matched the predicted patterns, confirming correct cloning. Ladders represent 1 kb DNA markers.
