# Supplementary material for: Antigen Format Determines Immunogenicity of AAV-Based SARS-CoV-2 Vaccines: Full-Length Spike Versus Truncated Subunits
Source: Vaccines (Basel). 2025 Nov 24;13(12):1187. doi: 10.3390/vaccines13121187 (PMC12737442; doi:10.3390/vaccines13121187)
Supplement: Supplementary file 1 [file vaccines-13-01187-s001.zip › vaccines-3927555-supplementary.pdf]

Supplementary information

Table S1. The following oligonucleotide primers were used for ligation-independent cloning (LIC) and colony PCR to generate the SARS-CoV-2 antigen-expressing constructs.

Name of the construct	Name of the primer	Primer sequences 5'-3'
pAAV-SARS1-4	Var1F	agacgctagactagcatgcgactcttcgcgatgtacgggccag
	Var1R	caattgttgttgttttgctgtccataaaaccgcccagtagaag
	VecVar1F	tatggacagcaaaacaacaacaattggatctgcgctgattttgt
	VecVar1R	gtcgcatgctagtctagcgtctaaacgcgt
pAAV-SARS5	Var5F	ATGGACCTGGAAGGCAAGCAGGg
	Var5VecR	CCCTGCTTGCCTTCCAGGTCCATC

Table S2. Primer Sequences for Sanger Sequencing.

Name 5.	Name of the primer	Primer sequences 5'-3'
pAAV-SARS1-5	Var1SeqF1	CTGGGCGTCTACTATCACA
	Var1SeqF2	CAGACCAGCAACTTCCG
	Var1SeqR1	CGCTGATGGTGAAGTTGGT
	Var1SeqR2	GTCCAGCCGCTTGTGAT
	Var1SeqR3	CTGGAGCGGTGGTGAAA
	Var1SeqF3	CGTCGTGAACATCCAGAAA
	Var1SeqR4	CGTCCTCGTCGAACTTGC
pAAV-SARS4	Var4SeqF1	GAGATGAAGTGCGGCAGA
	Var4SeqF2	CTGGAAATCCTGGACATCAC

Table S3. List of antibodies and reagents used in flow cytometry.

Antibody/Reagent	Manufacturer	Cat. No.
CD4-PC5.5	BD	332772
CD8-PC7	Biolegend	344713
CD62L-APC-A750	Biolegend	104449

CD44-KO525	Biolegend	103011
IFNγ-FITC	Biolegend	506504
TNFα-PB450	Biolegend	438204 (rat)<br>430904 (mouse)
IL2-PE	Biolegend	431807
Zombie Aqua	Biolegend	423101
CytoFix/CytoPerm intracellular staining kit	BD	554714
TruStain FcX (anti-mouse CD16/32)	Biolegend	101319

Table S4. Geometric Mean Titers of antibodies in immunized animal groups.

Group	Neutralization assay		ELISA_SARS-CoV-2		ELISA_AAV9	
	4 weeks	12 weeks	4 weeks	12 weeks	4 weeks	12 weeks
1	97.5[1,1,2]	640.0[2,3,4]	3901	28265	689[3]	312[6]
2	5.0[1]	59.4[2]	181	4755	95[3]	78[6]
3	5.0[1]	5.0[3]	221	362	689[4]	172[7]
4	5.0	5.0	100	110	14[4]	13[7]
5	6.7[2]	6.7[4]	221	328	464[5]	128[8]
6	5.0	5.0	100	100	20[5]	32[8]
7	5.0	5.0	100	100	8	8

Blue superscript numbers indicate statistically significant differences when comparing doses: [1,2,3,4,5,7,8] $p < 0.0001$; [6] $p = 0.0271$. Red superscript numbers indicate statistically significant differences when comparing vectors (high dose): [1,2,3,4] $p < 0.0001$.

Plasmid	Fragment length	Theoretical map	Result of restriction analysis
pAAV-SARS1	NotI+XhoI<br>177bp+2569bp+4899bp	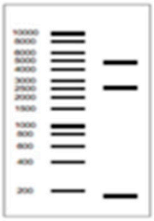	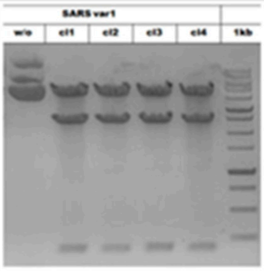
pAAV-SARS2	XhoI+<br>MluI<br>5069 + 2576	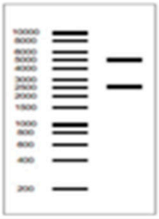	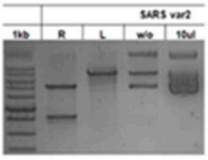
pAAV-SARS3	XhoI+<br>MluI<br>3290bp + 2576bp	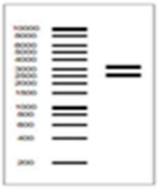	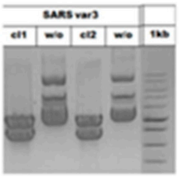
pAAV-SARS4	BglII+<br>MluI<br>1289 + 3725	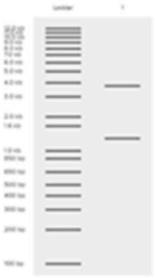	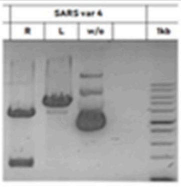
pAAV-SARS5	5010bp + 2576bp + 41bp	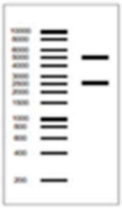	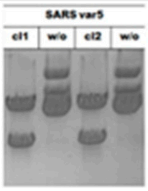

Figure S1. Validation of plasmid constructs by restriction analysis. Maps of the pAAV-SARS1-5 plasmids, theoretical restriction digest schemes, and corresponding agarose gel electrophoresis results. Plasmids were digested with specific restriction enzymes as indicated: pAAV-SARS1 – NheI/BbsI; pAAV-SARS2 and pAAV-SARS3 – MluI; pAAV-SARS4 – BstBI/MluI; pAAV-SARS5 – EcoRV. The observed fragment sizes matched the predicted patterns, confirming correct cloning. Ladders represent 1 kb DNA markers.
